# Opportunities and Limitations of Crop Phenotyping in Southern European Countries

**DOI:** 10.3389/fpls.2019.01125

**Published:** 2019-09-25

**Authors:** Joaquim Miguel Costa, Jorge Marques da Silva, Carla Pinheiro, Matilde Barón, Photini Mylona, Mauro Centritto, Matthew Haworth, Francesco Loreto, Baris Uzilday, Ismail Turkan, Maria Margarida Oliveira

**Affiliations:** ^1^LEAF, Instituto Superior de Agronomia, Universidade de Lisboa, Lisbon, Portugal; ^2^Biosystems and Integrative Sciences Institute (BioISI), Faculty of Sciences, Universidade de Lisboa, Lisbon, Portugal; ^3^FCT NOVA, Universidade Nova de Lisboa, Monte da Caparica, Portugal; ^4^ITQB NOVA, Universidade Nova de Lisboa, Oeiras, Portugal; ^5^Estación Experimental del Zaidín, Consejo Superior de Investigaciones Científicas (CSIC), Granada, Spain; ^6^HAO-DEMETER, Institute of Plant Breeding and Genetic Resources, Thermi, Greece; ^7^Institute for Sustainable Plant Protection, Italian National Research Council (IPSP-CNR), Sesto Fiorentino, Italy; ^8^Trees and Timber Institute, CNR, Sesto Fiorentino, Italy; ^9^Department of Biology, Agriculture and Food Sciences, CNR, Rome, Italy; ^10^Department of Biology, Faculty of Science, Ege University, I˙zmir, Turkey

**Keywords:** heat and water stress, crop selection and performance, high-throughput, phenotyping technology, phenotyping infrastructures

## Abstract

The Mediterranean climate is characterized by hot dry summers and frequent droughts. Mediterranean crops are frequently subjected to high evapotranspiration demands, soil water deficits, high temperatures, and photo-oxidative stress. These conditions will become more severe due to global warming which poses major challenges to the sustainability of the agricultural sector in Mediterranean countries. Selection of crop varieties adapted to future climatic conditions and more tolerant to extreme climatic events is urgently required. Plant phenotyping is a crucial approach to address these challenges. High-throughput plant phenotyping (HTPP) helps to monitor the performance of improved genotypes and is one of the most effective strategies to improve the sustainability of agricultural production. In spite of the remarkable progress in basic knowledge and technology of plant phenotyping, there are still several practical, financial, and political constraints to implement HTPP approaches in field and controlled conditions across the Mediterranean. The European panorama of phenotyping is heterogeneous and integration of phenotyping data across different scales and translation of “phytotron research” to the field, and from model species to crops, remain major challenges. Moreover, solutions specifically tailored to Mediterranean agriculture (e.g., crops and environmental stresses) are in high demand, as the region is vulnerable to climate change and to desertification processes. The specific phenotyping requirements of Mediterranean crops have not yet been fully identified. The high cost of HTPP infrastructures is a major limiting factor, though the limited availability of skilled personnel may also impair its implementation in Mediterranean countries. We propose that the lack of suitable phenotyping infrastructures is hindering the development of new Mediterranean agricultural varieties and will negatively affect future competitiveness of the agricultural sector. We provide an overview of the heterogeneous panorama of phenotyping within Mediterranean countries, describing the state of the art of agricultural production, breeding initiatives, and phenotyping capabilities in five countries: Italy, Greece, Portugal, Spain, and Turkey. We characterize some of the main impediments for development of plant phenotyping in those countries and identify strategies to overcome barriers and maximize the benefits of phenotyping and modeling approaches to Mediterranean agriculture and related sustainability.

## Introduction

### Major Agricultural Crops in Mediterranean Europe

Mediterranean countries are important producers of vegetables, legumes, cereals, fruits, olives and olive oil, grapes, and wine. Vegetable production accounts for 13.7% of the EU’s agricultural output with 63.9 million tons of vegetables grown in 2016 (EUROSTAT, 2017). Spain and Italy were the leading producers, contributing 24.1% and 17.4% of EU vegetable production, respectively. Tomatoes, carrots, and onions were the most widely produced vegetable crops. In 2016, 18 million tons of tomatoes were produced, 60% originating from Italy and Spain (EUROSTAT, 2017). Italy, Portugal, and Spain are also major centers for processing tomato and tomato paste production (Costa and Heuvelink, 2018). The largest EU fruit producers are Spain (33.4%), Italy (18.7%), and France (11.4%). Olives, grapes, apples, oranges, and peaches are the main components of EU fruit production and are mainly grown in Mediterranean countries such as Italy, Spain, Portugal, and Greece (Avni et al., 2017, EUROSTAT, 2017; OIV, 2019). Regarding processed grapes, the EU is the leading global wine producer, with about 50% of the world’s vine-growing area (3.2 million ha) and sustaining about 65% of wine production by volume (EUROSTAT, 2017; OIV, 2019). Spain, France, and Italy account for 81% of the cultivated area of grapevines within the EU. They are followed by Germany, Portugal, Romania, and Greece (EUROSTAT, 2017). The EU accounts for 75% of global olive oil production (10 million tons in 2016), and about 95% of the world’s cultivated area of olive trees is located in Mediterranean countries. Leading EU producers of olives are Spain (65.6%), Italy (19.4%), Greece (9.5%), and Portugal (4.8%) (EUROSTAT, 2017).

Total cereal production (rice included) in the EU reached 309 million tons in 2017, representing 12% of global production (EUROSTAT, 2018). According to the same source, wheat is the most cultivated cereal in EU, making up about 50% of total production. Of the remaining half, two-thirds correspond to maize and barley (EU Commission, 2019a). Other cereals grown in smaller quantities are rye, triticale, oats, and spelt. Cereal production represents 54% of arable land (55.5 million ha) within the EU and is economically important in Mediterranean countries, under irrigated and non-irrigated conditions. France has the greatest area devoted to cereal production with 9.5 million ha and is followed by Germany (6.3 million ha), Spain (6.0 million ha), and Italy (3.1 million ha) (EUROSTAT, 2018). In Greece and Portugal, cereals are also economically important, due to their resilience to drought under the harsh climatic conditions. Rice is also a significant crop in European countries such as Greece, Spain, Portugal, and Italy, where there are ongoing breeding programs. In contrast to wheat, which is largely exported from the EU, rice is mainly imported. Several breeding programs for cereals are being implemented in EU (e.g., WHEALBI—improving European wheat and barley production), targeting selection for more resilient cultivars to the demands imposed by climate change, as a result of greater attention from breeders, traders, and governments (Kahiluoto et al., 2019).

Outside the EU, Turkey is a major contributor to European and global agricultural production. In 2017, Turkey accounted for approximately 70% (12.6 million tons) of EU tomato production (TURKSTAT, 2018). Moreover, cereal production in Turkey accounts for approximately 50% of the country’s total arable area (38 million ha) with a production output of 34.5 million tons. The most important crops are wheat (20.6 million tons), barley (6.7 million tons), and maize (6.4 million tons) (TURKSTAT, 2018). Turkey is also a major global producer of fruits and vegetables (Güneş et al., 2017), specifically, grapes (4.2 million tons), apples (3 million tons), olives (2.1 million tons), oranges (1.9 million tons), hazelnuts (0.7 million tons), and green tea (1.3 million tons) (TURKSTAT, 2018).

Forestry is another economically crucial sector for Europe, in addition to providing environmental sustainability and key ecosystem services. European forests can also help to mitigate the impact of climate change and enable adaptation policies. Indeed, EU forests absorb the equivalent of about 10% of the total annual greenhouse gas emissions from the EU (EU Commission, 2019b). Forest species are thus another phenotyping target in a Mediterranean context, with its own needs and specifications (e.g., selection traits), but our focus will be on agricultural crops and related phenotyping issues in the Mediterranean.

### Challenges and Limitations in Mediterranean Agriculture

The Mediterranean region typically experiences long periods of drought and abrupt rainstorms and floods that negatively impact on crop performance as well as the available water and soil resources. The region has typical dry periods combined with high temperatures and a marked inter-annual weather variability, which results in a large and a highly variable demand for irrigation and in higher incidence of pests and diseases (Iglesias et al., 2012; Bebber et al., 2014; Giorgi, 2006; Giorgi and Lionello, 2008). Spain, France, Italy, Greece, and Portugal account for 86% of the total irrigated land in the EU (EU, 2015). Increased inter-annual weather variability makes it more difficult to predict drought periods and successfully implement mitigating measures (e.g., improved irrigation management). Decreased precipitation and extended drought spells tend to cause salt intrusion in coastal areas, reducing the quality of water resources and increasing soil degradation (EEA, 2012; IPCC, 2014; Hart et al., 2017; EASAC, 2018). Soil quality and health are obvious matters of concern worldwide (Bai et al., 2018; EASAC, 2018), and EU directives are focused on a more sustainable use of soil, water, fertilizers, and biocides. There is a wide variation in terms of Mediterranean agricultural systems, in the allocation of arable land percentage for each crop, farm size, and the area of farmland that is irrigated (**Supplementary Table 1**). The EU’s farming production system is based on small-sized farms, particularly in the Mediterranean (**Supplementary Table 1**), which poses additional challenges to the agro-food sector. As compared to larger enterprises, small agricultural operations are more susceptible to climate change and prone to economic variation due to limited funds and/or capacity to invest (Campbell and Thornton, 2014; Hart et al., 2017). In parallel, increased urbanization, excessive land fragmentation, climate change, and shortage of natural resources (e.g., water), all contribute to increased vulnerability in the Mediterranean region, specifically, Portugal, Greece and Turkey (**Supplementary Table 1**; Costa et al., 2016, Yucer et al., 2016).

A more comprehensive understanding of the specific agricultural environment found in the Mediterranean region is required to support rural development and to meet the goals of the EU-Common Agriculture Program of CAP 2014-2020 (EU, 2015; EU Commission, 2017a). More productive and sustainable agriculture depends on up-to-date knowledge and use of modern technologies, preferably at low cost, to enable more precise use of inputs and to promote circularity (EIP-AGRI, 2019). Technologies that improve data acquisition, interpretation, and management to enhance logistic organization in the food chain can help to boost EU agri-food supply chains (EU Commission, 2017b).

Digitization of agriculture and expansion of precision farming can reduce costs and minimize the environmental impact of agriculture (EU, 2015). For example, geo-location technologies could be used by farmers to comply with the Nitrates Directive, which concerns water protection against nitrates pollution from agricultural sources (EPRS, 2017). In parallel, novel agronomic strategies for canopy and soil management, alongside the development of genotypes that are more resilient to drought and heat stress, are in great demand from the Mediterranean agricultural sector (Mhadhbi et al., 2015; Zivy et al., 2015; Ollat et al., 2016, EPRS, 2017; Haworth et al., 2018a; Novara et al., 2018; Reynolds et al., 2019).

## Crop Phenotyping in the EU

### Different Scales and Approaches

Plant phenotyping, based on non-destructive image analysis, data management, and modeling, has emerged as a cutting-edge technology playing an important role in plant and agronomic sciences, namely, to design new crops, characterize the responses of genetic resources to the environment, and improve the breeding and management of crops (i.e., precision agriculture). Therefore, implementation of such technologies is crucial for sustainable agro-food operations, while optimizing the use of natural resources such as water and soil.

Investments in phenotyping facilities have taken place across the globe over the last decade including in Europe (see https://eppn2020.plant-phenotyping.eu/EPPN2020_installations#/). These high-throughput platforms are generating considerable volumes of data that provide valuable insights for plant/agronomic sciences and plant/crop breeding as evidenced by the increasing number of publications derived from these phenotyping facilities (Costa et al., 2019).

**Figure 1:**
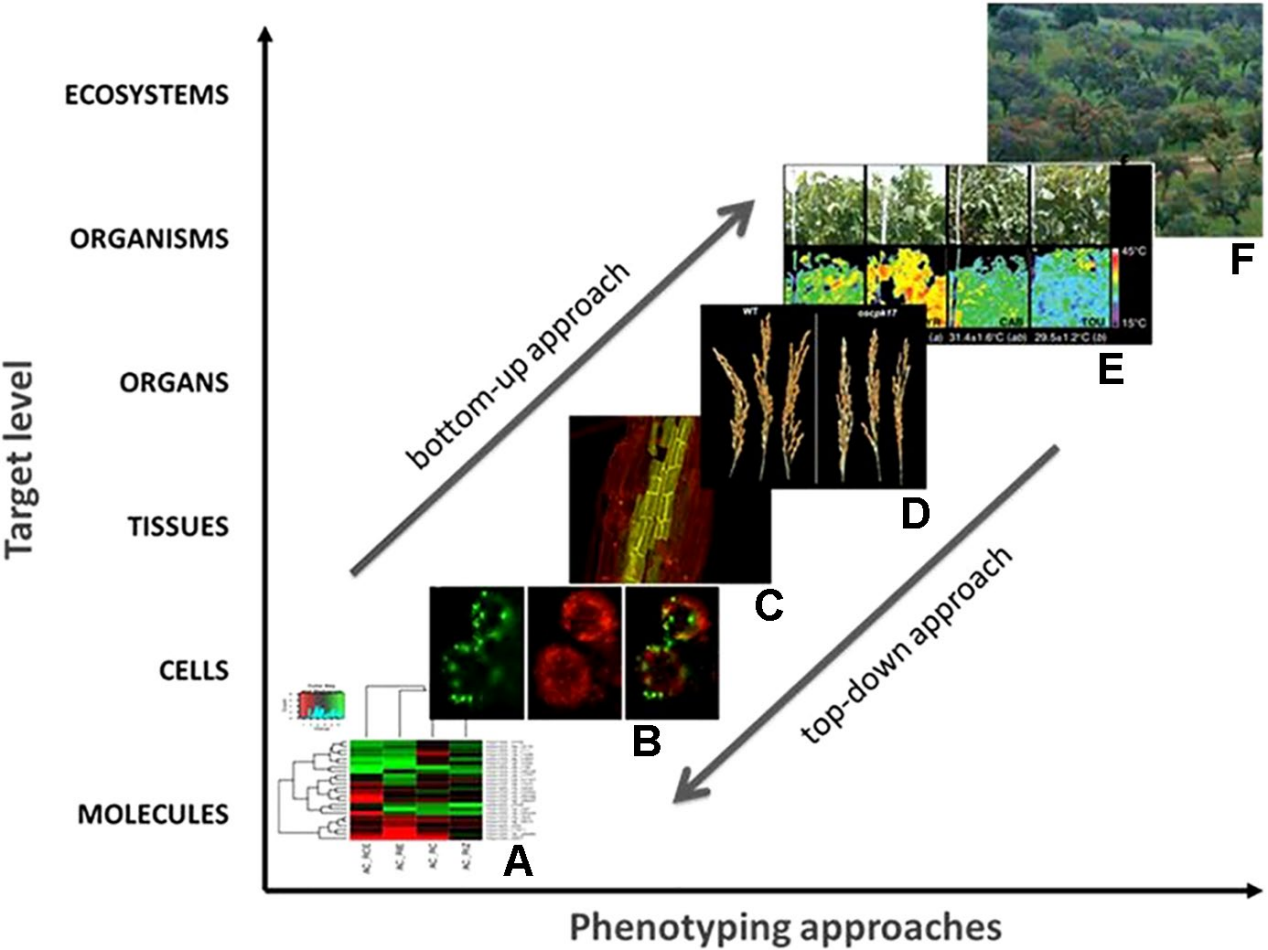
Phenotyping at different scales and levels of plant biological organization: from the molecular level **(A)** until the plant **(E)** and ecosystem level **(F)**. Arrows indicate the complementarity between different phenotyping levels and the need of integrating phenotyping outputs for improved practical application e.g., in crop selection and breeding. Different plant species were used as examples (*Quercus suber* L., *Fontinalis antipyretica* Hedw., *Arabidopsis thaliana* L., *Oryza sativa* L., and *Vitis vinifera* L.).

Plant phenotyping can be carried out at different levels of biological organization (**Figure 1**) with similar aims and outputs. At one extreme is molecular phenotyping (proteomics, metabolomics, etc.) and the related areas of cellular and tissue phenotyping (the latter less common in plants (Schiefelbein, 2015)), and at the other extreme is whole-plant/ecosystem phenotyping (**Figure 1**).

Organ phenotyping (e.g., leaves, roots, fruits) is most commonly used in high-throughput screening, with structural phenotyping (number of fruits, leaf area, leaf shape, leaf color, shoot elongation, plant architecture, etc.) and functional phenotyping (e.g., leaf gas exchange, volatile organic compounds, etc.) being investigated in both model species and crops (Costa et al., 2015; Ghanem et al., 2015; Walter et al., 2015; Tardieu et al., 2017; Reynolds et al., 2019). Top-down and bottom-up phenotyping approaches can be therefore designed, depending on research and application goals (**Figure 1**). Whole-plant phenotyping is closely related to breeding and biotechnology programs to deliver improved cultivars that are able to cope with environmental stresses. Molecular/cellular phenotyping is directed toward the investigation of gene function and biochemical pathways underpinning physiological mechanisms affecting development, productivity, and stress responses (Dhondt et al., 2013).

Phenotyping requires networks and interactions of sensor and data bases (e.g., new sensors, automation, machine learning, big data) with biological and agricultural sciences (integration of physiology with omics at different time and space scales in diverse environments). Phenotyping is also increasingly linked to modeling studies. Phenotyping should ideally be conducted at maximal throughput (number of units processed per unit time), resolution (distance between two spatial or time points), and dimensionality (number of captured traits). However, in practice, a trade-off between these three phenotyping descriptors must occur due to economic, technological, time, and personnel constraints (Dhondt et al., 2013). The integration of information across different levels of biological organization is impaired because of such trade-offs. Due to technological limitations, phenotyping at organ and cellular levels is undertaken at low throughput but (potentially) high dimensionality. In contrast, whole-plant phenotyping, is increasingly operated on a high-throughput but low dimensionality basis. To overcome the existing gap between the molecular and the whole-plant levels, we need increased dimensionality in high-throughput plant phenotyping (HTPP) alongside increased throughput in molecular and cellular phenotyping. The establishment of shared plant phenotypic ontologies (Coppens et al., 2017) will produce “computable phenotypes” (Deans et al., 2015) and progresses in modeling will facilitate data and knowledge integration (e.g., Tisné et al., 2008; van Eeuwijk et al., 2019).

To support and accelerate plant improvement, plant phenotyping must be based on more cost-effective technologies and sensors (Reynolds et al., 2019; Roitsch et al., 2019). In this context, remote-sensing methods based on imaging are increasingly used in multiple applications: (a) eco-physiology studies and crop breeding (Costa et al., 2013; Deery et al., 2014; Sankaran et al., 2015), (b) decision-making for precision agriculture (Zhang and Kovacs, 2012; Costa et al., 2013; Gago et al., 2017), (c) yield prediction (Son et al., 2013), and (d) prediction of spatial field variability in experimental sites (Araus and Cairns, 2014; Zaman-Allah et al., 2015). Remote-sensing techniques are non-destructive, non-invasive, fast, cost-efficient, and well-correlated with several agronomical and physiological crop traits (Marino et al., 2014; Reynolds et al., 2015; Condorelli et al., 2018). Moreover, remote sensing can provide an idea of spatial variability through time. Cheaper approaches (e.g., unmanned aerial vehicle [UAV]–based imaging technologies) can support field-phenotyping trials in plant-breeding programs, but simpler and more cost-effective image analysis and processing are required (Holman et al., 2016; Santesteban et al., 2017). The role of modeling as a tool to integrate knowledge on crop phenotyping is also crucial. Integrating phenotyping and modeling approaches can help to screen and identify complex traits in plants e.g., transpiration efficiency in cereals (Chenu et al., 2018) or in grapevine (Gago et al., 2017).

### Plant Phenotyping in Europe: A Heterogeneous Panorama

Plant phenotyping remains largely heterogeneous in Europe, with a greater investment in facilities in countries such as France, Germany, or the Netherlands (**Table 1**), followed by lower levels in Spain, Portugal, Greece, and Turkey. Developments regarding infrastructure, technology, knowledge, and training and application have been occurring at different speeds and intensities when we compare Northern and Southern EU countries. In particular, budget limitations commonly experienced by research and academic institutions in Southern European countries may preclude construction of plant-phenotyping infrastructures. Plant phenotyping in Europe is also heterogeneous in regards to the requirements of phenotyping (field *vs*. greenhouse crops, traits of interest) (see **Table 1**). Technological developments in precision and digital farming, alongside crop-phenotyping and phenomics, could provide valuable information regarding crop/cultivar adaptability to abiotic and biotic stress targeted toward more sustainable crop production (Araus and Cairns, 2014; Araus et al., 2018; Reynolds et al., 2019). Yet, due to the high costs in the short term, establishment of farm-based phenotyping infrastructures is unachievable for many EU countries. The economic crisis, the generally small size of companies involved in this sector (i.e., breeding), lack of perception by growers/officials, and delayed public and private investments in phenotyping infrastructures have limited plant-phenotyping capabilities in Southern Europe. The lack of pilot-phenotyping infrastructures in public institutions and universities makes it more difficult to identify and meet the technological challenges associated with precision breeding and farming systems, as well as restricting public awareness about the advantages of phenotyping infrastructures. Within Mediterranean countries, France and, to a lesser extent, Italy remain as exceptions (**Table 1**). France and Germany are pioneers in plant phenotyping, with a higher number of facilities and publication output in this domain (Costa et al., 2019). In 2018, the French Phenotyping Network (www.phenome-fppn.fr/) congregated nine phenotyping platforms, including controlled conditions (two units), semi-controlled conditions (two units), field conditions (three units), and molecular platforms (two units). This panorama contrasts with other Southern Mediterranean countries (**Table 1**).

**Table 1:** Summary of the major characteristics of phenotyping activities developed in EU countries analyzed in this study (Portugal, Spain, Italy, and Greece) and in Turkey. Data on French, Dutch, and German situations is provided as examples of advanced levels of phenotyping approaches/technology and to enable a more robust comparison between Northern and Southern EU countries.

Country	National network	Inter-national Networks	Field and indoor phenotyping	Funding	Main crops	Future
Portugal	None*	COST FA1306 EMPHASIS (support country)	Molecular-level Chl fluorescence, IRGA,IR imaging, hyperspectral	Public (FCT), EU-funding No private funding	Fruit crops	Low-cost precision agriculture and breeding
Spain	None*	COST FA1306 EMPHASIS (support country)	Molecular-level Chl and multicolor fluorescence, IRGA, IR imaging Hyperspectral HTPP (indoor)	Public (Central and regional Governments) EU-funding	Fruit crops, vegetables Model plants	Low-cost precision agriculture and breeding
Greece	None*	COST FA1306 EMPHASIS (support country)	Molecular level at indoor phenotyping	EU-funding	Fruit crops	Field-phenotyping facility for research and breeding. Low-cost precision farming
Italy	PHEN-ITALY (13 research organizations, Universities, and regional agencies)	COST FA1306 EMPHASIS (core country) EPPN	Molecular-level HTPP (indoor) Chl fluorescence, IRGA, IR imaging Hyperspectral	Public (CNR, ALSIA) EU-funding Private	Cereals and fruit crops	Low-cost precision agriculture and breeding
Turkey	None	COST FA1306	Molecular level at indoor phenotyping Hyperspectral	Public (TUBITAK) EU-funding Private	Cereals and fruit crops	Low-cost precision agriculture and breeding
France	French Plant Phenotyping Network (www.phenome-emphasis.fr/)	COST FA1306 EMPHASIS (core country) EPPN (core member)	Molecular-level HTPP (outdoor and indoor) Chl fluorescence IRGA, IR imaging Hyperspectral	Public EU-funding Private	Cereals, fruit crops, grapevine, vegetables	Low- and high-cost phenotyping, precision agriculture and breeding
Germany	German Plant Phenotyping Network (https://dppn.plant-phenotyping-network.de)	COST FA1306 EMPHASIS (core country) EPPN (core member)	Molecular-level HTPP (outdoor and indoor)	Public EU-funding Private	Field and greenhouse crops	Low- and high-cost phenotyping, precision agriculture, and breeding
The Netherlands	Netherlands Plant Eco-Phenotyping Centre (www.npec.nl) PhenomicsNL (www.phenomics.nl)	COST FA1306 EMPHASIS (core country) EPPN (core member)	HTPP (outdoor and indoor)	Public EU-funding Private	Field and greenhouse crops (e.g., vegetables, ornamentals)	Low- and high-cost phenotyping, precision agriculture and breeding

*These countries are represented at EMPHASIS via EMPHASIS support group; ALSIA, Agenzia Lucana di Sviluppo e di Innovazione in Agricoltura; CNR, National Research Council of Italy; FCT, Portuguese National Science and Technology Funding Institution; TUBITAK, Scientific and Technological Research Council of Turkey; Chl, chlorophyll; HTPP, high-throughput plant phenotyping; IRGA, infrared gas analyzer; IR, infrared.

**Figure 2:**
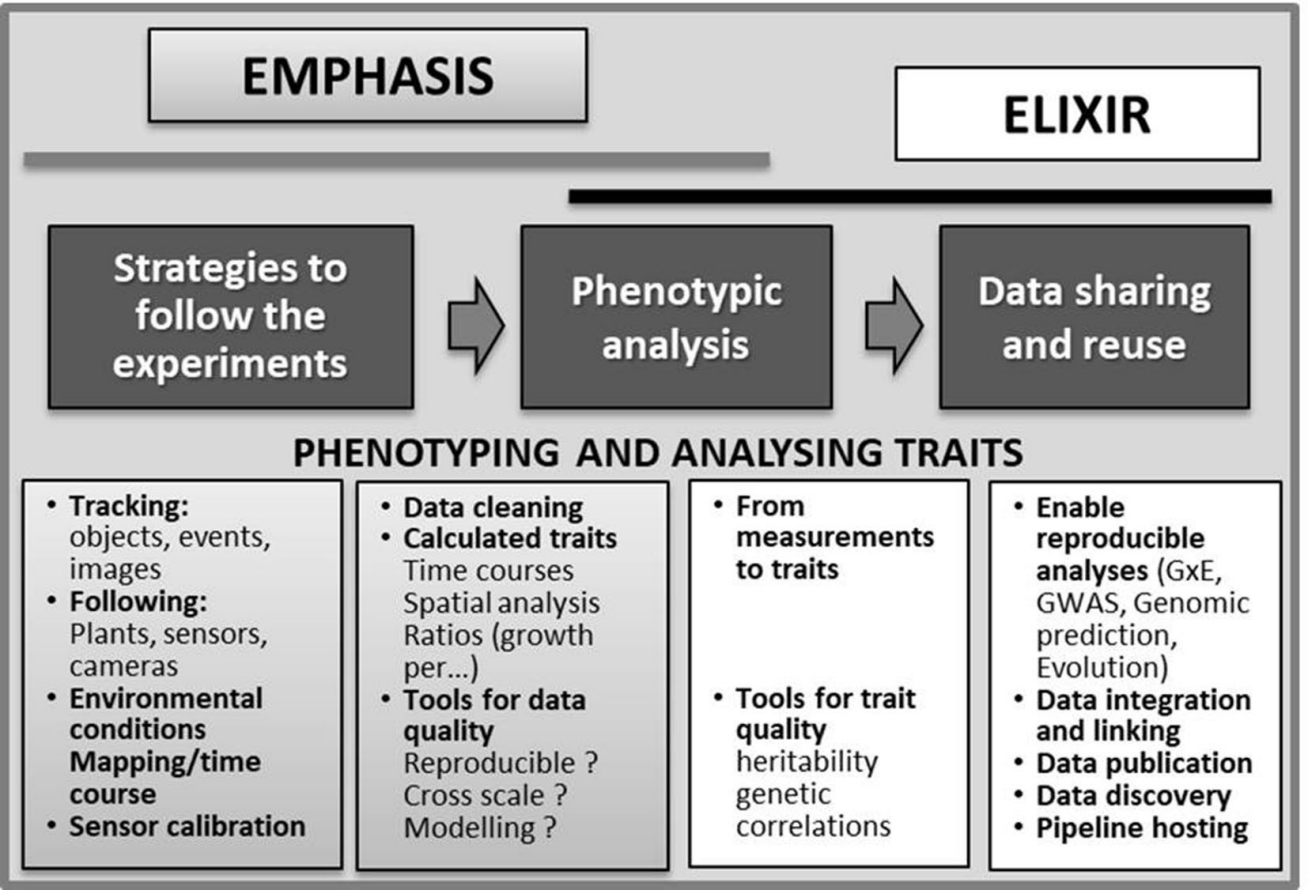
Data flow from trials to genetic analyses and dissemination considered within the EMPHASIS and ELIXIR which are two ESFRI infrastructures, dedicated to phenomics and data sharing and integration, respectively. ESFRI is the European Strategy Forum on Research Infrastructures, aiming at developing scientific integration of Europe and to strengthen its international outreach. The relation between data production/analysis and modeling will be promoted by the interaction of the academic/industrial communities focused on phenomics (e.g., EMPHASIS) as well on crop modeling (e.g., AgMIP project—Agricultural Model Intercomparison and Improvement Project, http://www.agmip.org/) (adapted from EMPHASIS, 2018).

Improved integration of phenotyping and genetic and genomic platforms and resources should facilitate increased understanding of crop performance for the development of more effective breeding strategies and also guarantee improved big data management (Lopes et al., 2014; Coppens et al., 2017; Reynolds et al., 2019). To accomplish these aims within the EU, the European Plant Phenotyping Network (EPPN) (supported by the H2020 Programme) has been created. It will provide the European public and private scientific sectors access to a wide range of modern plant-phenotyping equipment and methodologies and promote efficient exploitation of genetic resources for crop improvement (http://www.plant-phenotyping-network.eu/eppn). The EPPN established a pan-European infrastructure that includes the whole phenotyping pipeline (involving sensors and imaging techniques), data analysis, and interpretation in a biological context and meta-analyses of experiments carried out on different organs and at different scales of plant organization. The EPPN currently operates in 10 countries providing access to 36 different plant-phenotyping facilities to the users. Furthermore, the European Strategic Forum for Research Infrastructure (ESFRI) has recognized plant phenotyping as a priority for the European research area to achieve food security in a changing climate context. Consequently, EMPHASIS (European Infrastructure for Multi-Scale Plant Phenomics and Simulation—https://emphasis.plant-phenotyping.eu) was included in the ESFRI Roadmap 2016 (https://www.esfri.eu/roadmap-2016) as a project to develop and implement pan-European plant phenotyping facilities available to the user community. EMPHASIS aims at increasing the integration of major players and users involved in data acquisition, management, storage, and sharing, to provide a more practical use of the available phenotyping data to analyze genotype performance in diverse environments (EMPHASIS, 2018). EMPHASIS is organized to permit fair access to phenotypic, environmental, and meta-analysis data sets for genetic analysis and modeling. This process is currently under development in the EMPHASIS PREP and EPPN2020 projects (EMPHASIS, 2018, **Figure 2**). The EU COST Action FA1306 (*The quest for tolerant varieties—phenotyping at plant and cellular level*) has further contributed to improve networking by facilitating the exchange of researchers, knowledge, skills, and training across countries, thus leveraging the absence of phenotyping facilities within some EU countries.

To further enhance the sustainability of agriculture in Mediterranean countries, the establishment of phenotyping facilities remains a timely and indispensable issue. In the following section, we provide a short overview of the major characteristics of the existing phenotyping infrastructure and the knowledge and networks available in Southern Mediterranean countries.

## Plant Phenotyping in the South European Mediterranean Region

### The Situation of Greece and Portugal

Greece and Portugal share rather similar constraints in terms of agricultural production, related to climate change vulnerability and limited investments in innovation and technology. The recent economic crisis had a marked impact on the development and use of modern technologies in the agriculture and agri-food sectors. Therefore, the panorama for plant/crop phenotyping is closely identical in the two countries and will be discussed in parallel.

Greek agriculture is based on a large number of annual and perennial crops including grains, pulses, vegetables, fiber, and olive and fruit orchards. A significant number of local landraces are present and cultivated in islands and marginal lands. During the last 50 years, breeding efforts and programs provided an increased number of registered varieties. Plant breeding in Greece is achieved mainly by conventional methodologies, because acquisition of molecular technologies was initiated recently. Consequently, phenotyping target traits such as leaf gas exchange, chlorophyll *a* (chl *a*) fluorescence, nutrient deficiencies, and infections have most commonly been assessed (Yol et al., 2015) by visual observations in combination with a range of chemical analyses. Nonetheless, modern technologies are being currently incorporated in breeding and to monitor crop performance. Significant efforts have been made in utilizing modern technologies to improve agricultural competitiveness (Koutsos and Menexes, 2019). In this respect, the emerging digitization of the Greek agricultural sector targets enhanced sustainable use of resources and inputs. The use of precision farming technologies can help to maximize farming output, in association with the use of the EU’s Copernicus system data by allowing farmers to receive warnings of extreme weather events and specific information on crop management (e.g., irrigation, fertilization). Several companies and associations are already providing digitized services to farmers for open field crops in Greece (Barnes et al., 2019), but the engagement of both the academic and research communities is crucial to train agronomists and scientists in the use of new technologies for data collection and processing.

For most crops in Portugal, selection and breeding work remains modest. However, similar to many other Mediterranean countries, Portugal is rich in ancient landraces and crop wild relatives that are relevant for breeding programs and germplasm collections. For instance, yield and quality traits of grapevine varieties and respective clones are currently under field evaluation, exploiting the large Portuguese collection of ancient grapevine varieties (30,000 genotypes held by the Portuguese Association for Grapevine Diversity, PORVID) (Gonçalves and Martins, 2012; Martins and Gonçalves, 2015). In grain legumes, a breeding program started in 1986, and 30 years of activity have resulted in 16 new varieties of pulses registered in the Portuguese National Catalogue of Varieties. The breeding program has contributed to an increase in the area of cultivated land in Portugal. As a result, highly adapted cultivars such as the chickpea “Elvar” have become the predominant chickpea cultivar in Portugal and southern France (I. Duarte, INIAV, pers. comm.). An intensive breeding program is also going on for rice (Negrão et al., 2013; Pires et al., 2015; Almeida et al., 2017) and other cereals; pastures and fodders, are also being targeted by the National Institute of Agrarian and Veterinary Research (INIAV).

In Portugal, phenotyping research targets are focused on both model species and crops, at the molecular/cellular level and at the whole-plant level. However, it is still predominantly low-throughput phenotyping, based for example on leaf gas exchange (Cruz de Carvalho et al., 2011; Ribeiro et al., 2016) or thermography (Costa et al., 2016) and medium-throughput phenotyping utilizing rapid chl *a* fluorescence induction transients (Silvestre et al., 2014). Portugal lacks a phenotyping network and HTPP infrastructures capable of supporting rapid speed characterization of relevant crop traits (e.g., growth, yield, quality, stress resilience). Such HTPP infrastructures should result from a joint effort of academic institutions and private agribusiness partners. Growers and officials have limited perception of the relevance of such infrastructures for improving crop productivity and harvest quality. Although the EPPN provides fully controlled environment facilities, phenotyping trials performed in climate-specific locations (in open field facilities) could increase complementarity between laboratory-based work and “real-world” scenarios. These facilities should preferentially be located in different bioclimatic Mediterranean zones to increase the coverage of more extreme conditions (e.g., high temperature trials). Therefore, investment in Mediterranean region countries is needed at three major levels:

(1) open-field facilities to study the eco-physiological dynamics of plant species and specific crops in their natural environment (this is relevant for grapevine, olive, legumes, cereals, and forest species);
(2) semi-controlled climate conditions within modern glasshouse facilities equipped with both contact and remote-sensing phenotyping tools to assess crop physiological performance;
(3) fully controlled climate conditions (climate chamber facilities) for molecular phenotyping (e.g., identification of key genes related to crop developmental traits and response/adaptation to stress); these facilities would complement the existing EPPN network, thus increasing the number of European facilities with fully controlled environmental conditions (in the entire EPPN network, only two facilities are currently able to perform heat stress experiments).

To optimize experimental design of trials and data analysis/processing, a web-linked computational facility for data management and modeling should be installed in parallel; this would also require standardization of the procedures used in data acquisition. Modern phenotyping procedures would increase the characterization of important crops, such as grapevine, where research currently extends from molecular physiology to crop eco-physiology and agronomy, including precision viticulture and remote sensing. Nonetheless, machine learning techniques alongside spectroscopic approaches are currently employed in *Vitis* phenotyping (Gameiro et al., 2016; Fernandes et al., 2018). The wine sector could particularly profit given the large inter and intra-varietal collections of *Vitis vinifera* L. available (Martins and Gonçalves, 2015) and the fact that the Iberian Peninsula is considered a secondary centre of grapevine domestication (Cunha et al., 2015). Improved conservation and characterization of germplasm collections would empower the competitiveness of Portuguese and Mediterranean viticultures. Tools to quantify the phenotypic impact of intra-varietal genetic variability and the means to carry out large-scale selection/breeding in both greenhouse and field conditions are required. Likewise, improved phenotyping facilities and tools at greenhouse and field levels for fruit, cereals (e.g., rice, maize), and legume crops (e.g., beans, chickpea) would assist in the characterization of regional genotypes/landraces and in the identification of putative adaptations to low-input farming systems, thus supporting the development of varieties with improved biotic and abiotic stress resistances.

For Greece and Portugal, the exploitation of current plant phenomics and crop-phenotyping technologies and strategies could provide valuable data for improved management of biodiversity resources, fostering crop/cultivar adaptability to climate and resistance against pests and diseases. However, field-phenotyping infrastructures are still unviable due to high costs. Some initiatives have been instigated to implement the use of phenotyping strategies in crop management, particularly using thermal and multispectral imaging alongside ground-based and aerial approaches (e.g. see projects INTERPHENO http://interpheno.rd.ciencias.ulisboa.pt/, WineClimAdapt or, VINBOT http://vinbot.eu/). For both Portugal and Greece, low-cost phenotyping approaches (and/or platforms) would be the most feasible way to move forward on the short to medium term, considering current financial constraints.

### The Situation of Spain

Spanish agriculture is highly competitive at both the European and global levels. The country is a major exporter of both fresh and processed agricultural and horticultural goods/products (MAPA, 2018). Nevertheless, drought and heat stress alongside increasingly vulnerable soil water resources due to climate change make the present scenario more negative especially for Southern provinces (AEMET, 2018). Spanish agriculture and horticulture have, over the last decades, experienced large investments in innovation and technology, specifically directed to irrigation and modern management systems (precision farming, remote sensing, etc.) (Giannakis et al., 2016; Lainez et al., 2018). The financial support of both central and regional governments to the agribusiness has favored the competitiveness of Spanish agriculture/horticulture (Alarcón and Arias, 2018; https://europa.eu/european-union/topics/agriculture_en).

Spanish research groups are working at the whole-plant-phenotyping level mainly through the use of imaging techniques for stress detection at the lab and field levels. Laboratory-based phenotyping research in Spain has been focused on biotic plant stress (parasitic plants and pathogens e.g., viruses, fungi and bacteria) using low-throughput imaging sensors for chl *a* fluorescence, UV induced-multicolor fluorescence (MCF: blue, green, red, and far-red), and thermography (Pineda et al., 2011; Pérez-Bueno et al., 2015; Pineda et al., 2017; Ortiz-Bustos et al., 2017). Some groups are focused on field-phenotyping approaches and precision agriculture, stress detection, and irrigation management by making use of hyperspectral and thermal sensors at the field and plant levels (Calderón et al., 2013; López-López et al., 2016; Zarco-Tejada et al., 2016; Gago et al., 2017).

Plant and ecosystem functions are also evaluated by mean of proximal and remote sensing, as well as diverse ecophysiological measurements (Gamon et al., 2016; Zhang et al., 2017). This research is focused on climate change, atmospheric pollution, and emission of biogenic volatile organic compounds (VOCs). Plant phenotyping methods are used in breeding programs and to improve the understanding of the ecophysiological and metabolic mechanisms of abiotic stresses responses in Mediterranean crops. Phenotyping technology has been tested in both controlled and field conditions. For example, the performance of UAV and field-based HTPP approaches have been compared using RGB and multispectral and thermal aerial imagery in combination with ground-based sensors to assess varieties of barley and maize under different nitrogen and phosphorus treatments (Araus and Cairns, 2014; Gracia-Romero et al., 2017; Kefauver et al., 2017).

The relationship between photosynthesis and production under drought conditions in Mediterranean crops is of special interest. The production of high-quality wines in Protected Designation of Origin Areas under a full range of climate conditions is particularly important in Spain (Resco et al., 2016). In grapevine studies, UAV technology using thermal sensors has been employed to monitor the physiological status of plants subjected to different irrigation treatments alongside ground-based physiological stress measurements at the leaf-and-stem level (Gago et al., 2017). Researchers and private wine cellars from different wine-producing regions of Spain are working together with companies specialized in robotic research. The main goals of this collaboration are the application of unmanned mobile robots (aerial and ground vehicles) to optimize precision farming to reduce the input of phytosanitary products in vineyards and help farmers in decision-making.

The use of phenotyping techniques in the early detection of emerging pathogens is of particular interest in Spain and other South European countries. *Xylella fastidiosa* is a quarantine pathogen in the EU, and it has been detected in Spain, Italy, France, and Germany (https://www.eppo.int/ACTIVITIES/plant_quarantine/shortnotes_qps/shortnotes_xylella). *X. fastidiosa* is a threat to multiple crops (e.g., the olive, almond trees, grapevine, citrus, and ornamentals) across the whole Mediterranean (Olmo et al., 2017; Zarco-Tejada et al., 2018). Phenotyping tools (e.g., thermography and multispectral sensors) can help to rapidly assess symptoms and identify novel resistant cultivars while supporting controlling measures of the pathogen (Parisi et al., 2019).

The economic crisis had, and still has, a major impact on public investment in research in Spain (Alarcón and Arias, 2018). Similar to Portugal and Greece, financial constraints may explain the lack of HTPP platforms in Spain. Therefore, research efforts have been mainly directed in the development of low-cost phenotyping strategies and establishment of synergies in the country to create interdisciplinary teams/networks, combining expertise in robotics, imaging sensors, big data, plant physiology, breeding, and precision farming. Integration of Spanish phenotyping research within the existing European Networks is needed. For example, no Spanish group has yet joined the EPPN, although some research groups are present in the EMPHASIS-PREP project that is preparing the coordination of the EU plant-phenotyping infrastructures and programs.

### The Situation in Italy

Research in Italy has focused on phenotyping for selection of crop and fruit tree varieties with improved tolerance to environmental stresses, in particular heat stress and drought (e.g., Lauteri et al., 2014; Chakhchar et al., 2017; Haworth et al., 2018b). However, interest in different applications of plant phenotyping has also emerged recently. For example, the impact of higher winter temperatures on impaired dormancy and yield in high-value fruit trees, such as almonds (Hoeberichts et al., 2017), and the development of drought tolerant biomass crops for cultivation on rain-fed marginal lands that are not currently utilized for food production (e.g., Haworth et al., 2017) have formed the basis for phenotyping studies. The majority of this phenotyping work has involved “low-cost” or “low-throughput” methodologies such as biometry, photosynthetic gas exchange, and carbon isotope discrimination (ခδ^13^C) analyses (e.g., Centritto et al., 2009; Haworth et al., 2018b). However, automated screening (Minervini et al., 2014) and more in-depth biochemical and genetic investigations within controlled environment facilities have commenced and are working in collaboration with key phenotyping infrastructures in Europe (Danzi et al., 2019). The on-going goal for phenotyping in Italy and much of Europe is to be able to characterize the phenome of large numbers of individuals, including those for which the genetic bases have been investigated, under conditions representative of current and future challenges to plant growth and production.

**Figure 3:**
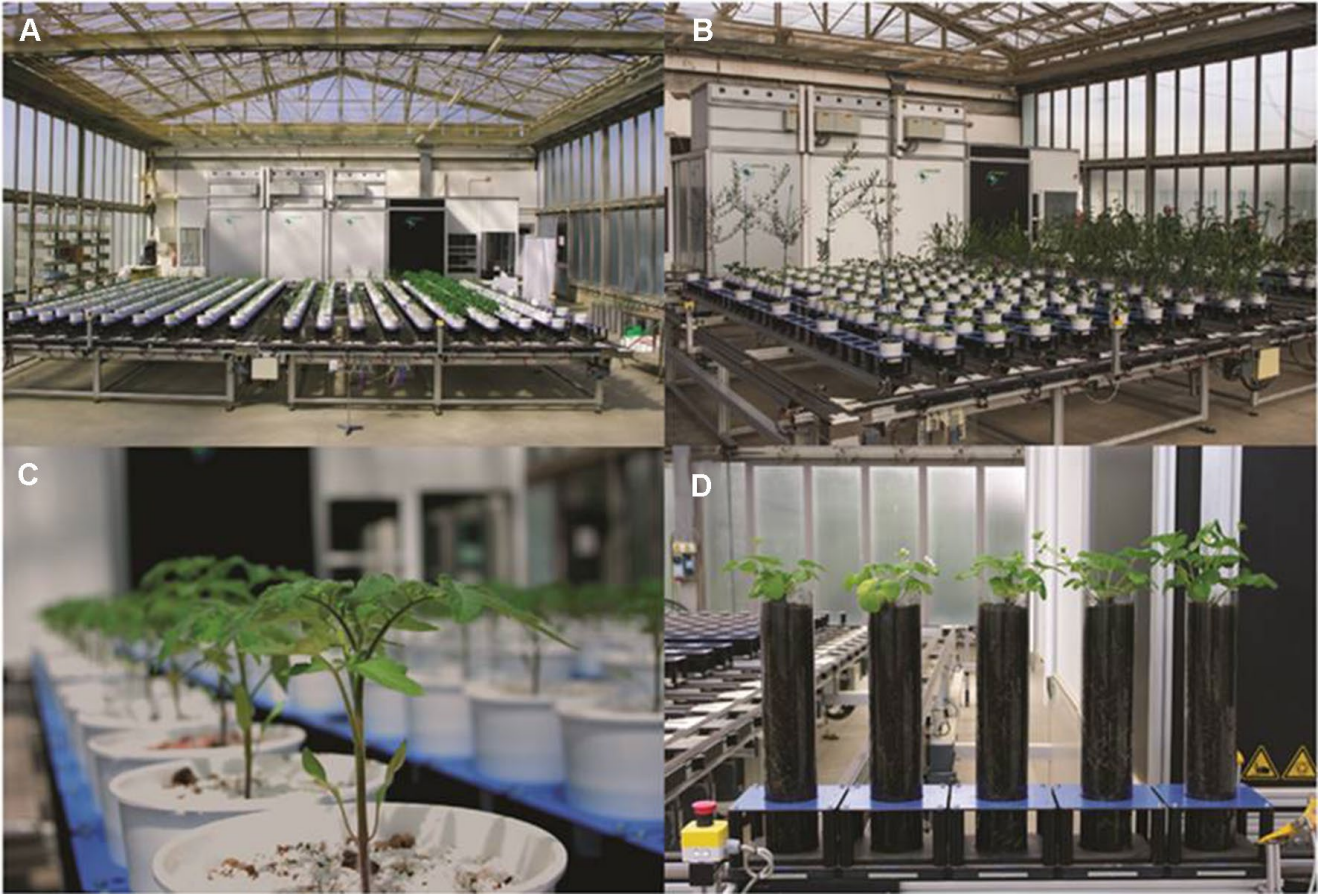
The ALSIA greenhouse phenotyping facility (Basilicata, southern Italy) consisting of Scanalyzer 3D-systems which include conveyor belts (0.3 km and 500 cars) and image chambers: **(A**, **B)** the robot system for randomization of pots, consistent monitoring of pot weight, accurate application of water and imaging of plants; **(C)** phenotyping of tomato seedlings; and **(D)** phenotyping of root architecture.

Policy makers and researchers in Italy have acknowledged the necessity to address the challenges posed by the delay in matching current progress in genotyping and breeding with adequate phenotyping for assisted selection. There is wide political and scientific consensus that developing the capacity and capability of plant phenotyping will be a formidable tool in mitigating the impact of climate change and land degradation, and their consequences on agricultural production and the overall farming economy. The Italian Plant Phenotyping Network (PHEN-ITALY: www.phen-italy.it) was established in 2017 to coordinate and develop plant-phenotyping research within Italy. The PHEN-ITALY network consists of 13 research organizations, universities, and regional agencies led by the National Research Council of Italy (CNR) and Agenzia Lucana di Sviluppo e di Innovazione in Agricoltura (ALSIA) where a large HTPP infrastructure is operating. CNR is a partner within the EMPHASIS-PREP. PHEN-ITALY aims to become the Italian infrastructural node of EMPHASIS. PHEN-ITALY also participates in the EPPN and the activities and projects of the International Plant Phenotyping Network (IPPN). Following the main principles of plant phenotyping (non-invasive and high-throughput measurements), the PHEN-ITALY infrastructure is based on optical systems (LemnaTec GmbH Scanalyzer Laboratory and Greenhouse models) that include a conveyor system, two imaging stations (fluorescence, NIR, and RGB sensors), and three computer stations to study: (i) the phenotypic background of genetically characterized crops of interest in Italy (durum wheat, tomato), (ii) the root to shoot architecture of plants, and (iii) the VOCs emitted by plants as markers of biodiversity and stress. For example, 3D scanning and chlorophyll fluorescence technology have been used to non-destructively assess the physiological and molecular responses to drought of tomato sprayed with a commercial mixture of amino acids and nutrients (Petrozza et al., 2014) and genetic differences in nitrogen assimilation in *Lotus japonicus* (Parlati et al., 2017). Moreover, PHEN-ITALY supports public–private partnerships focusing on the development of innovative diagnostic and analytic technologies based on the interaction of plant genes, nutrition, and environmental stress (**Figure 3**) (Santaniello et al., 2017).

These collaborations between publically funded research organizations and commercial partners will enhance the applicability of phenotyping to “real-world” scenarios through enhanced genetic and molecular crop development. The identification and development of crop varieties that are not only tolerant to stress, but with high nutritional quality, can ensure the future economic and ecological viabilities of European agriculture. In Italy, the value of precision agriculture has been recognized by allocation of national funds to phenotyping technologies as part of the trans-disciplinary drive toward “digital agriculture.” Increased automation and digital scanning technologies will facilitate non-invasive HTPP (Minervini et al., 2014; Scharr et al., 2016). In conjunction with transcriptomic and gene expression analysis, these HTPP screening approaches can be developed to remove the “phenotyping bottleneck” and enable a greater understanding of the GxExM interaction of genes (G), their environment (E), and the management (farming) practices (M) crop experience. Diverse collections of food (for example, the extensive CNR olive collection at the IVALSA Santa Paolina Azienda Sperimentale, Follonica, Tuscany—GPS coordinates: 42°56’02’’N 10°45’52’’E—which is made up of over 1,000 genotypes) and biomass (for example, collections of perennial grasses at the Universities of Catania and Bologna—Cosentino et al., 2006; Valli et al., 2017) crop varieties will provide platforms to develop “innovative facilities” where traditional phenotyping technologies can be integrated with novel approaches such as online analysis of the VOCs emitted during abiotic and biotic stress (i.e., VOC phenotyping), or remote spectroradiometry conducted by the growing number of unmanned aerial vehicles. The combination of expertise and biological resources available in Italy will maximize the potential of phenotyping technologies to address many of the challenges facing Mediterranean agriculture.

### The Situation of Turkey—A Non-EU Member

Although there has been a major effort to adopt modern agronomic practices and technologies in Turkey, the low education levels of many farmers act as a barrier to disseminate such technologies. Plant phenotyping in Turkey is mostly based on quantitative description of anatomical, physiological, and biochemical properties of plants (Demirel et al., 2014; Kahriman et al., 2016; Kandemir et al., 2017). Turkey is not keeping pace with rapid developments in the field of automated, non-destructive image analysis or sensor-based phenotyping, and in most cases, phenotyping is performed manually. It is therefore difficult to undertake phenotyping in a high-throughput manner, especially at the physiological and biochemical levels. In addition, a national framework is needed to increase cooperation between scientists working in the field (determination of traits, yield, and performance) and those working in the lab (cellular- and molecular-level phenotyping). This is insufficient at present. Phenotyping is usually performed by agricultural research institutes funded by the government and private sectors to select breeding material. In addition, universities undertake phenotyping in basic and applied research for breeding purposes; none of which is high-throughput.

There are some individual efforts to adopt and/or develop new phenotyping technologies in Turkey that are limited to academia (e.g., Kurucu et al., 2004; Genc et al., 2011; Ustuner et al., 2014; Kahriman et al., 2016; Ünal et al., 2017). In recent years, the main scientific funding agency in Turkey, the Scientific and Technological Research Council of Turkey (TUBITAK), announced grant calls to support selection of new breeding materials and development of new cultivars for various crop species, which provides an opportunity to establish facilities for HTPP. However, another bottleneck for the use of high-tech phenotyping techniques in Turkey is insufficient human resources, especially people educated in software and computer engineering willing to work with and assist plant biologists. Therefore, strategies and plans to provide sufficient skilled personnel must be adopted by the government, universities, and private sector. To solve these problems and to explain the need of HTPP approaches to both public and private institutions, a national phenotyping network should be established to support individual efforts within the country. Turkey would gain additional benefit from HTPP due to its large genetic diversity. The south eastern region of Turkey is known as the “fertile crescent,” where the early domestication of many crop plants occurred (Abbo et al., 2010). For example, archaeological findings suggest that einkorn wheat (*Triticum monococcum*) was grown around Sanliurfa between 10,000 and 12,000 years ago (Kilian et al., 2007).

Due to its rich diversity in landraces and wild relatives of crop plants, Turkey has great potential to contribute toward the development of abiotic or biotic stress–resistant crops. However, this would be only possible by utilization of HTPP techniques to elucidate new traits and catalogue phenotypes of these genotypes.

## Discussion

The increasing constraints imposed by declining water quality/quantity, soil erosion, loss of soil fertility, and climate change with the associated increase in the incidence of extreme events poses significant strategic challenges for Mediterranean agriculture. This fosters the need of the agricultural sector to develop new agronomic practices and select novel varieties/cultivars for crops and orchards. Plant phenotyping has emerged as an indispensable tool to support precision agriculture and crop breeding for the benefit of crop production and agricultural development. In fact, plant phenotyping will increasingly play a vital role in the sustainability of Mediterranean agriculture in the context of climate change and scarcity of resources such as soil and water. Identification and development of novel crop varieties resilient to heat and drought stresses will help to minimize the agronomic, ecological, and socio-economic vulnerabilities to climate change in the Mediterranean region (e.g., Killi et al., 2016; Fraga et al., 2018). Therefore, it is of the outmost importance to improve the coordination of plant-phenotyping activities in Mediterranean countries to deliver the greatest benefit in terms of value and research outputs for the region. In addition, phenotyping may also enhance education and technological innovation, providing employment opportunities for highly skilled professionals.

### Integrating Information Across Biological Levels

The ability to integrate information across different levels of organization (molecules, cells, tissues, organs, whole plants, canopies) is crucial to understand crop/plant responses to the environment, but such integrated information remains comparatively limited. Moreover, we must identify which traits to phenotype, and at which moment of the plant’s life cycle this should be done in a fast but robust way. These major questions must be addressed along with more specific issues during any investigation. The lack of integrated phenotyping data is a general drawback in plant phenotyping but is of particular importance in the Mediterranean region and related agriculture due to the structural complexity of field cropping systems (e.g., size and architecture of vineyards, olive trees, and orchards). The use of non-invasive methods facilitates more objective and repeatable measurements of traits such as plant architecture, fruit quality, and/or the impact of biotic or abiotic stress under field conditions. Moreover, improvements in phenotyping imaging technology are resolving limitations associated with variable backgrounds and light conditions (Kicherer et al., 2017). To effectively integrate complex information from contrasting biological organization levels, an increase in the dimensionality of HTPP is required alongside an increased capacity for molecular and cellular phenotyping. Large-scale investments in research capacity and staff training are thus needed. Phenotyping of Mediterranean crops could also be enhanced by increased knowledge of crop physiology. Currently, several EU Mediterranean countries such as Portugal, Spain, Greece, and Italy (as well as countries outside EU, like Turkey) integrate within international networks that provide access to phenotyping facilities established in other EU countries and also benefit from specific support in networking and expertise (EPPN, EMPHASIS, COST).

Other relevant topics are data handling and modeling to accurately describe complex traits relevant to plant productivity and responses to biotic and abiotic stresses. New advanced bioinformatics tools for data integration are required to process large sets of complex data produced through phenotyping pipelines to generate new knowledge on crop phenotypic plasticity in response to climate change. Meanwhile, and in contrast to experimental platforms, models have unlimited access and can be run independently by users. To this extent, the EMPHASIS infrastructure is able to provide the data/model quality policy that facilitates articulation between experimental platforms and models (https://emphasis.plant-phenotyping.eu/modelling). HTPP platforms and tools offer a new avenue in the parameterization of models to scale up data from molecular and morphological levels to breeding. Integration of phenotyping data with crop growth models can provide the means to evaluate and quantify the usefulness of agronomic practices [e.g., irrigation, (bio-)fertilizer, pesticide, etc.] to optimize resource use, as well as generate genotypes for different environmental contexts such as resilience to climate change. To this end, different classes of G2P (genotype-to-phenotype) models have been shown to help predict complex phenotypic traits as functions of GxExM interaction (van Eeuwijk et al., 2019).

### Overcoming Barriers

The EPPN already provides both field and greenhouse facilities to assess crop performance in the Northern climate zone of Europe. Breeding programs for Mediterranean agriculture (and horticulture) demand improved selection criteria and phenotyping strategies for both field and greenhouse conditions. To achieve this, we must: (1) define selection criteria for crop and phenotyping traits, according to the specific breeding needs of each country/region; (2) optimize the practical implementation of phenotyping efforts, as a function of the specific conditions of each country; (3) raise financial support among public and private stakeholders, and (4) include HTPP in national research policies for agriculture.

Increasing integration of information across HTPP facilities (indoor, greenhouse or field) or different biological levels (as depicted in **Figure 1**) is crucial for more efficient phenotyping (Coppens et al., 2017). In parallel, it also allows improved data analysis/processing and enhanced modeling capabilities by combining different types of expertise.

The use of field-phenotyping technologies to monitor plant/crop responses should be expanded to enhance assessment of larger numbers of varieties/replicates under natural growth conditions at a lower cost. Moreover, successful phenotyping strongly depends on the recruitment of multidisciplinary teams involved in research programs and projects, especially where there is still a lack of skills and educational background for phenotyping issues. This demands time and investment in training in Mediterranean countries.

Each country should identify the main developers and users of infrastructures, the main benefits, and the beneficiaries. This would guarantee a more effective allocation of national and EU funding and available resources as well as a more efficient and sustainable operation and maintenance of such infrastructures. This requires political awareness and support because it depends on financial resources and investment.

Within the EU, a major aim should be improved training and support through COST actions focused on crop phenotyping. This has shown excellent results in the promotion of networking, between academia, companies, and governments on different phenotyping topics, as well as providing short-term training opportunities for researchers, and thus bridging the gaps that may possibly appear due to the heterogeneity in training and facilities described in this review.

Data-driven strategies can help the breeding industry to define better strategies for selection and using fewer resources. Developing models to identify robust patterns of experimental data may help breeders and agronomists to more accurately choose individuals with improved productivity. More active involvement of the private sector can also develop phenotyping strategies in Mediterranean countries. The contribution of these companies can be useful to support educational programs in the use of big data for agriculture.

More integrated political action is required at different levels (local, regional, national, and European) to bring together farmers, the agro-industry, government, and consumers to identify the most adequate strategies to face the adversities associated with climate change in Mediterranean countries (Aguiar et al., 2018). The roles of local, regional, and central governments can be crucial to support investments related to the adaptation of agriculture to climate change and crop phenotyping.

### Conclusions and Prospects

The rapid (and expensive) development of phenotyping technologies can partly explain the heterogeneous panorama of phenotyping infrastructure within Europe. Indeed, differences in the establishment of infrastructure, budget, knowledge, and technological capacities are apparent when comparing Northern and Southern European countries. There are further differences amongst Mediterranean countries as well. The major ones are apparent in the available infrastructure, availability of qualified labor, dimension of the companies (breeders, phenotyping technologies), and priorities of national and international networks. Solutions to the lack of financial resources, a problem common to Mediterranean countries (Portugal, Greece, and Turkey) are needed. Public–private partnerships could assist in resolving this lack of investment and foster political will. This public–private approach is already being implemented in some countries through the close cooperation of academic research institutes and commercial-breeding companies.

The establishment of phenotyping and phenomics facilities and infrastructures in Southern EU member states is crucial to meet the EU 2020 targets of smart specialization, sustainable growth, and the development of an inclusive and circular economy. Particularly in light of the budget limitations, commonly experienced by Southern European countries, phenotyping infrastructures should rely on simple, lower cost, and rapid methods, which are user-friendly, reliable, and robust. Improved phenotyping approaches will enhance our understanding of crop responses to Mediterranean climate conditions (combinations of high air/soil temperature, drought, increased CO_2_, etc.). In this way, it will be possible to properly assist breeding and selection of genotypes/phenotypes better suited to the prevailing and forthcoming climatic conditions in the Mediterranean. In parallel, the development of image processing, analysis, and modeling is also crucial to ensure relevant outputs. Interdisciplinary education of young researchers and technicians, from both public and private sectors, will also benefit from the establishment of phenotyping infrastructures.

Improved networking to avoid excessive duplication of EU phenotyping facilities must be guaranteed to ensure that phenotyping facilities are complementary. However, Mediterranean countries will not solve their regional problems by only making use of facilities installed elsewhere. Phenotyping of Mediterranean crops must take place where breeding is performed and in the environments where they are/will be cultivated if adaptation to climatic variables is the target. Therefore, the investments and facilities that exist in different countries must be analyzed, so that efficient EU policies may be designed to secure EU funding for Mediterranean countries, to serve the phenotyping needs and preserve the technological sovereignty of member states. In parallel, efforts should be directed to develop low-cost phenotyping strategies and promote synergies between different Mediterranean countries by creating transnational and interdisciplinary teams or networks that combine expertise in robotics, imaging, big data, plant/crop eco-physiology, and genetics.

We urge policy makers in the Mediterranean region to promote investments in plant phenotyping to cope with ongoing and predicted climate change. Plant phenotyping can play a central role in climate change adaptation and mitigation strategies in the Mediterranean region. Partnerships between public research organizations and the private sector (including breeding or technology companies) should be encouraged to implement phenotyping technologies in agricultural and climate change scenarios and to further incorporate genetic and molecular approaches to improve crop productivity.

## Author Contributions

All authors wrote the paper and read and approved the final manuscript.

## Conflict of Interest Statement

The authors declare that the research was conducted in the absence of any commercial or financial relationships that could be construed as a potential conflict of interest.
