# Supplementary material for: Opportunities and Limitations of Crop Phenotyping in Southern European Countries
Source: Front Plant Sci. 2019 Sep 25;10:1125. doi: 10.3389/fpls.2019.01125 (PMC6774291; doi:10.3389/fpls.2019.01125)
Supplement: Supplementary file 1 [file Table_1.docx]

**Supplementary Table 1. Detailed statistics on EU’s agriculture farming and forest system, based on data from the EUROSTAT, TURKSTAT and the World Bank**

|  | **Greece** | | **Spain** | | **France** | | **Italy** | | **Portugal** | | **EU-28** | | **Turkey** | |  |
| --- | --- | --- | --- | --- | --- | --- | --- | --- | --- | --- | --- | --- | --- | --- | --- |
| Total area (km^2^) ^(a, b)^ | 131912 | | 498504 | | 549060 | | 301291 | | 88847 | | 4369364 | | 785562 | |  |
| Cropland (% of tot. area) ^(a, c)^ | 15,3 | | 21,3 | | 28,9 | | 25,1 | | 11,7 | | 22,2 | | 48,30 | |  |
| Grassland (% of total area) ^(a, c)^ | | 19,4 | 19,0 | | 26,7 | | 21,7 | | 23,6 | | 20,7 | | 18,60 | |  |
| Wood & shrubland (% of total area) ^(a)^ | | 56,7 | 45,7 | | 33,8 | | 39,5 | | 52,8 | | 44,8 | | - | |  |
| Number farm holdings(x 10^3^)^(e)^ | | 710 | 965 | | 472 | | 1010 | | 264 | | 10841 | | - | |  |
| % Very small & small farms (< 20 ha) ^(e)^ | | 95,2 | 78,6 | | 42,8 | | 87,1 | | 91,3 | | 86,3 | | - | |  |
| % large farms (> 100 ha) ^(e)^ | | 0,14 | 5,39 | | 20,76 | | 1,49 | | 2,27 | | 3,11 | | - | |  |
| Used Agricultural Area (UAA) (x 10^6^ ha) ^(e, c)^ | | 3,38 | 21,69 | | 27,06 | | 11,82 | | 3,54 | | 165,65 | | 15,53 | |  |
| % UAA per country ^(e, c)^ | | 25,62 | 43,51 | | 49,28 | | 39,23 | | 39,84 | | 37,91 | | 19,78 | |  |
| % UAA managed by high-input farms ^(e)^ | | 27,9 | 15,2 | | 53,5 | | 27,0 | | 9,1 | | 31,8 | | - | |  |
| % UAA managed by medium-input farms ^(e)^ | | 35,2 | 21,7 | | 32,7 | | 25,1 | | 8,6 | | 28,9 | | - | |  |
| % UAA managed by low-input farms ^(e)^ | | 36,9 | 63,1 | | 13,8 | | 47,9 | | 82,3 | | 39,3 | | - | |  |
| Irrigable area (% of UAA) ^(e,d)^ | | 44,9 | 31,1 | | 10,4 | | 33,9 | | 15,6 | | 11,3 | | 13,60 | |  |
| Irrigated area (% of UAA) ^(e, c)^ | | 24,0 | | 12,4 | | 5,1 | | 23,7 | | 13,1 | | 6,2 | | 34,70 | |

**(a)** data for 2015; EUROSTAT explorer dataset (lan_lcv_ovw); **(b)** data for 2015 and for Turkey at Eurostat explorer dataset (cpc_agmain); **(c)** data for Turkey at TURKSTAT (2018), http://www.turkstat.gov.tr/UstMenu.do?metod = temelist; **(d)** data for Turkey - World Bank World Development Indicators for the same year as EU countries; **(e)** data for 2013; Agriculture and fisheries Statistical books, EU 2016 (ISBN 978-92-79-63350-8) https://ec.europa.eu/eurostat/documents/3217494/7777899/KS-FK-16-001-EN-N.pdf/cae3c56f-53e2-404a-9e9e-fb5f57ab49e3; **(f)** data for Turkey at EUROSTAT explorer dataset (cpc_agmain) for the same year as EU countries.
